# Prognostic significance of the infiltration of CD163^+^ macrophages combined with CD66b^+^ neutrophils in gastric cancer

**DOI:** 10.1002/cam4.1420

**Published:** 2018-03-24

**Authors:** Xiaopei Huang, Yamin Pan, Jun Ma, Zhengchun Kang, Xiaowen Xu, Yan Zhu, Jikuai Chen, Wei Zhang, Wenjun Chang, Jiangbo Zhu

**Affiliations:** ^1^ Department of Health Toxicology Second Military Medical University Shanghai 200433 China; ^2^ Department of Environmental Hygiene Second Military Medical University Shanghai 200433 China; ^3^ The First Department of Endoscopy Shuguang Hospital Shanghai University of Traditional Chinese Medicine Shanghai 201203 China; ^4^ Department of Colorectal Surgery Changhai Hospital Second Military Medical University Shanghai 200433 China; ^5^ Department of Pathology Changhai Hospital Second Military Medical University Shanghai 200433 China

**Keywords:** Gastric cancer, immune microenvironment, macrophages, neutrophils, prognosis

## Abstract

The polarization of tumor‐associated macrophages (TAMs) and tumor‐associated neutrophils (TANs), especially from the antitumoral phenotype to the protumoral phenotype under certain conditions, has an important influence on the progression of tumors. However, the interactions and combined prognosis of these cells are poorly known. Here, we detected the infiltration of CD68^+^ TAMs, CD163^+^ TAMs, and CD66b^+^ TANs in the specimens from 662 patients with GC by immunohistochemistry. The results showed that the infiltration of each of CD163^+^, CD68^+^, and CD66b^+^ cells in GC tissue was significantly increased and independently associated with GC prognosis. Strong collinearity (r = 0.690, *P *<* *0.001) was found between the infiltration of CD163^+^ and CD68^+^ cells in GC, and multivariate Cox analysis confirmed the infiltration of CD163^+^ cells was a better predictor for prognosis than that of CD68^+^ cells. The combination of the infiltration of CD163^+^ and CD66b^+^ cells provided more accurate survival prediction than any individual marker. Patient subgroups with CD66b^low^CD163^low^ (hazard ratio (HR) = 2.161; 95% confidence interval (CI)  = 1.266–3.688; *P *<* *0.001), CD66b^high^CD163^high^ (HR = 3.575; 95% CI = 2.155–5.933; *P *<* *0.001), and CD66b^low^CD163^high^ (HR = 7.514; 95% CI = 4.583–12.312; *P *<* *0.001) were gradually associated with shorter DFS when compared with the subgroup with CD66b^high^CD163^low^. The similar result was also for DSS among the subgroups. Moreover, the two‐marker model could more effectively discriminate the prognosis among the patients with chemotherapy than that among those without chemotherapy. We concluded that CD163^+^ TAMs were a more valuable prognostic marker than CD68^+^ TAMs, and CD163^+^ TAMs combined with CD66b^+^ TANs could more precisely predict the prognosis of patients with GC.

## Introduction

Gastric cancer (GC) is estimated to be the fifth most common malignancy worldwide 1. Despite the great advancement in diagnosis and treatment modalities, the prognosis of patients with GC remains poor due to tumor recurrence and metastasis 2. Accurate assessment of the prognosis is helpful in choosing the most appropriate and timely treatment for patients with GC. The tumor node metastasis (TNM) staging system is widely used as a prognostic model by clinicians at present. However, the staging system could not provide full prognostic information, as even patients with the same TNM stage tumor might have markedly different prognosis. Therefore, there is an urgent need for a precise classification of GC that can be used to better predict prognosis of patients.

GC is an inflammatory disease and frequently characterized by the infiltration of a markedly heterogeneous polynuclear and mononuclear cells containing macrophages, granulocytes, and various subpopulations of T lymphocyte 3, 4, 5. Of these, the versatility and plasticity of immune cells polarization, especially tumor‐associated macrophages (TAMs) and tumor‐associated neutrophils (TANs), have turned to be essential in the tumor progression. For one thing, TAMs participate in the tissue homeostasis and repair. For another, they are involved in promoting tumor progression through remodeling the extracellular matrix (ECM), enhancing tumor cell migration and invasion, and modulating angiogenesis 6, 7. Emerging evidence also suggests that neutrophils, in response to signals derived from cancer cells or stromal cells, can alter their phenotypes and migration routes and release factors that act on tumor cells 8, 9, 10. However, the interaction of these factors and the corresponding clinical significance in GC remain largely unknown. Recent studies have shown the likelihood that TANs could recruit TAMs precursors to the tumor site and promote the M2‐like activation of macrophages 11. Conversely, macrophages may resolve the inflammation response induced by neutrophils through the removal of the dribs of neutrophils 12. Moreover, previous studies have shown that TAMs and TANs are associated with the prognoses for patients with GC 13, 14. Thus, the incorporation of these two immunological parameters into the TNM staging system may add some prognostic value to further stratify and better manage patients with different prognosis.

CD68 was typically used as a marker of macrophages including both M1 and M2 macrophages. However, CD68 has wide expression in normal and neoplastic cells, making this protein unspecific to the monocyte/macrophage lineage 15. CD163, a hemoglobin/haptoglobin complex scavenger receptor, expressed almost exclusively on circulating monocytes and tissue macrophages, has been recognized as a valuable specific marker of M2 TAMs 16. CD66b is a highly glycosylated CEA family protein encoded by the CGM6 gene, which can be used to identify neutrophils and has been used in many tumors to identify TANs, including renal cell carcinoma, liver cancer, and GC 17, 18, 19. In this study, we evaluated the infiltration of immune cells marked with CD68, CD163, and CD66b in GC by immunohistochemical examination and focused on the combined prognosis of the latter two, hoping to precisely predict the prognosis and provide clues for the stratification treatment of patients with GC.

## Materials and Methods

### Patients

Formalin‐fixed, paraffin‐embedded (FFPE) tissues containing 662 cancer and 69 pericarcinomatous lesions were obtained from 662 patients with GC who underwent surgical resection at the Changhai Hospital during December 2006 and July 2011. Patients who had received preoperative chemotherapy or radiotherapy and those diagnosed with autoimmune diseases were excluded from the study. The clinical characteristics of each patient were collected, including age, gender, tumor size, differentiation status, TNM stage (according to the American Joint Committee on Cancer Staging Manual 7th edition), adjuvant chemotherapy, and serum CEA and CA199 levels. The postoperative follow‐up was performed at our outpatient clinic annually for an additional 5 years or until patient death. Disease‐free survival (DFS) was defined as months from the date of receiving surgery to the time of tumor relapse or metastasis. Disease‐specific survival (DSS) was defined as months from the date of receiving surgery to the time of death due to GC. This study was approved by the institutional review boards of Changhai Hospital. All patients were provided written informed consent.

### Immunohistochemistry

Tissue microarrays (TMAs) containing the FFPE specimens were commercially constructed by a specialized company (Outdo Biotech, Shanghai, China) and subsequently used to perform the immunohistochemistry examination with a standard protocol. Briefly, all array slides with a thickness of 3–4 μm were first deparaffinized using xylene and rehydrated using graded ethanol. Then, the slides were immersed and boiled in sodium citrate (pH 6.0) for 30 min in a pressure cooker for antigen retrieval. The endogenous peroxidase was inhibited by 3% H_2_O_2_ for 30 min. Primary antibodies against CD66b (dilution 1:400, no. 555723, BD Biosciences, NJ), CD68 (dilution 1:150, no. 955, Abcam, Cambridge, UK), and CD163 (Ready‐to‐use, MAB‐0206, Maxim, Fuzhou, China) were used to incubate the slides overnight at 4℃. Subsequently, the slides were treated with secondary antibody (MaxvisionTM2 HRP‐Polymer anti‐Mouse/Rabbit IHC Kit) for 10 min at room temperature. After washing with phosphate‐buffered saline (PBS), the slides were reacted with 3‐3′‐diamino‐benzidine (DAB) solution for 1.5 min and counterstained with hematoxylin for 30 secs. The manipulation was performed strictly in accordance with the experimental procedure.

### Quantitative evaluation of immunostaining

Stained TMA slides were digitally scanned using an Aperio ScanScope (Aperio Technologies, Vista, California, America) at a resolution of 40× bright field, and Aperio ImageScope software was used to view the images for analysis. The quantitative evaluation of immunostaining was performed separately by two experienced pathologists (CW and ZY) who were blinded to the clinical data. For each sample, five high‐power fields with the most homogeneous infiltrating immune cells in each 1‐mm^2^ field were evaluated as cell populations marked with CD163, CD68, or CD66b. Positive‐staining cells in all fields were counted manually, and the average count for each sample was recorded. Disagreements were resolved by consensus. Tumors were scored as “low” or “high” when the counts of CD163^+^ TAMs, CD68^+^ TAMs, and CD66b^+^ TANs were below or above the median value of the scores for all GC specimens.

### Statistical analysis

All analyses were performed using SPSS 19.0 for Windows (SPSS, Chicago, IL). A paired samples t‐test was used to compare the infiltration of CD163^+^, CD68^+^ TAMs, and CD66b^+^ TANs in GC tissues with those in the adjacent normal tissues. Pearson's correlation methods were performed to identify correlations for quantitative variables with normal distributions. Chi‐square and Mann–Whitney U tests were used to determine the associations between clinicopathological variables and the infiltrating condition (high or low) of each sample. Kaplan–Meier analysis with log‐rank test was performed to estimate DFS and DSS. Univariate and multivariate Cox regression analyses were used to identify independent prognostic factors. The results were considered statistically significant if *P *<* *0.05.

## Results

### Pattern and correlation of the infiltration of TAMs and TANs in GC

**Figure 1:**
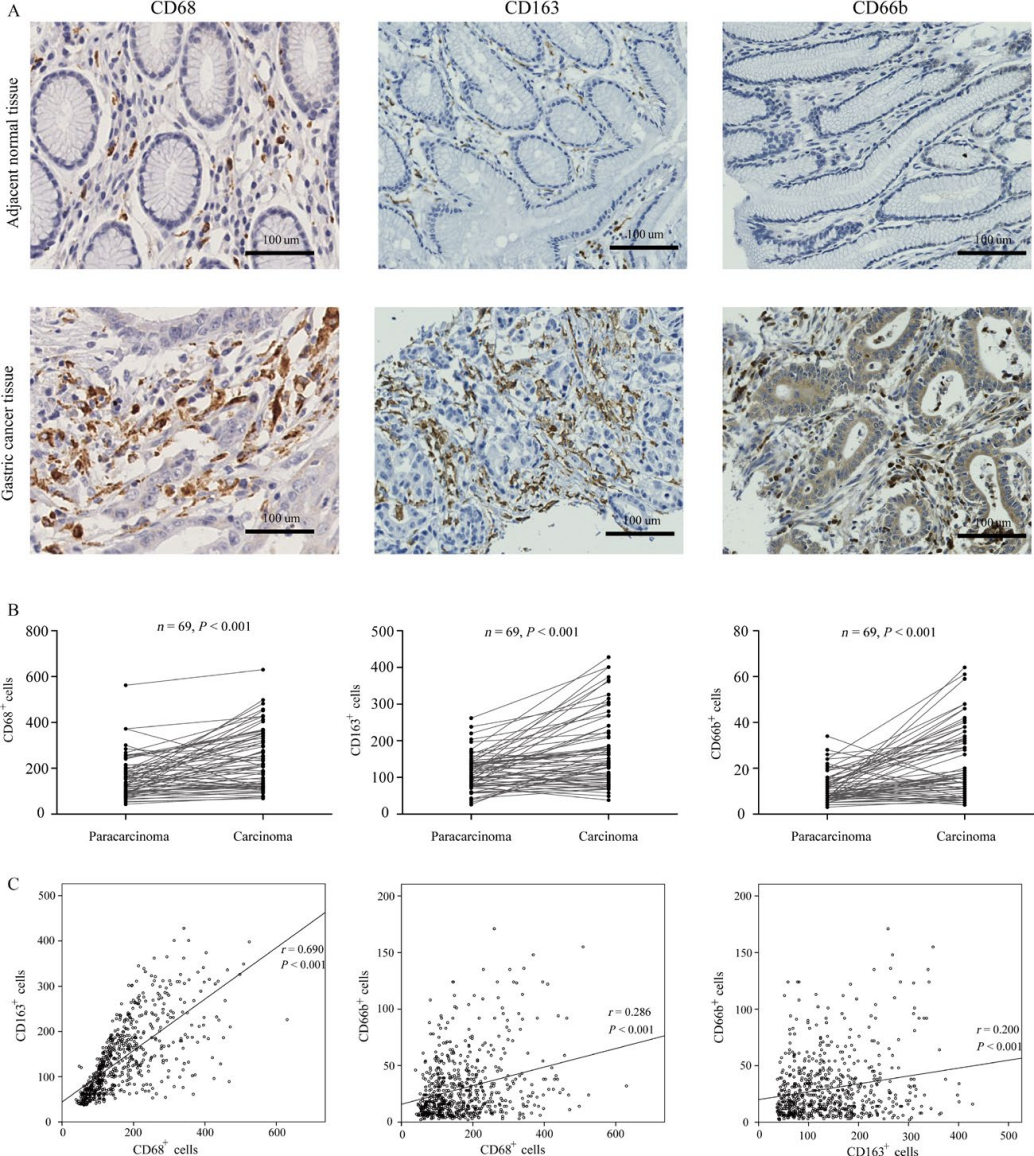
High infiltration of CD68^+^, CD163^+^, and CD66b^+^ cells and the correlations of these cell populations in GC. (A) Representative immunohistochemical images of the infiltration of CD68^+^, CD163^+^, and CD66b^+^ cells in GC and adjacent mucosa. (B) Elevated infiltration of CD68^+^, CD163^+^, or CD66b^+^ cells in GC. (C) Correlations of the infiltration of CD68^+^, CD163^+^, and CD66b^+^ cells in GC.

Immunoreactivity of antibodies targeting CD68, CD163, and CD66b was observed only in the gastric stroma, consistent with previous studies 14, 20, 21. The representative images of the infiltration of CD163^+^, CD68^+^, and CD66b^+^ cells in GC and adjacent mucosa are shown in Figure 1A. The results showed that the infiltration of CD163^+^, CD68^+^, and CD66b^+^ cells in GC was significantly higher than that in the adjacent mucosa (all *P *<* *0.001) based on 69 cancer–normal paired tissue samples (Fig. 1B). Among all 662 GC specimens, Pearson's correlation analysis was performed to identify correlations of the infiltration of different immune cells marked with CD163, CD68, or CD66b. As expected, the number of CD163^+^ TAMs was strongly positively correlated with CD68^+^ TAMs (r = 0.690, *P *<* *0.001) (Fig. 1C), and the number of CD68^+^ cells was significantly higher than that of CD163^+^ cells in these specimens, likely because antibody targeting CD68 lacks complete specificity for cells of the monocyte/macrophage system as previously mentioned 15. The number of CD163^+^ M2 macrophages accounted for about 88 percent (median) of CD68^+^ total macrophages. As shown in Figure 1C, only weakly positive correlations were found between the number of infiltrating CD163^+^ TAMs and CD66b^+^ TANs (r = 0.200, *P *<* *0.001) and between CD68^+^ TAMs and CD66b^+^ TANs (r = 0.286, *P *<* *0.001), which indicated that CD66b^+^ TANs may have different contributions to GC progression compared to TAMs.

### Associations between infiltrating immune cells and patient features

Patients with GC were first classified into the subgroups with high or low immune cell infiltration according to the median number of infiltrating CD68^+^, CD163^+^, or CD66b^+^ cells in 662 GC specimens. Then, the associations between the infiltration of each of CD68^+^ TAMs, CD163^+^ TAMs, and CD66b^+^ TANs and clinicopathological features were analyzed. As shown in Table 1, high infiltration of CD68^+^ or CD163^+^ TAMs was consistently and significantly related to old age, large tumor size, advanced TNM stage, and adjuvant chemotherapy (all *P *<* *0.050). Moreover, high infiltration of CD68^+^ and CD163^+^ TAMs was also significantly associated with poor differentiation (*P *<* *0.001) and high serum CEA levels (*P* = 0.003), respectively. Conversely, high infiltration of CD66b^+^ TANs was significantly correlated with small tumor size, well differentiations, and early TNM stage (Table 1). These results indicated that high infiltration of CD68^+^ or CD163^+^ macrophages was associated with aggressive characteristics of GC, whereas CD66b^+^ neutrophils in GC primarily showed the activities of antitumors.

**Table 1:** Association between the infiltration of immune cells and clinicopathologic features in gastric cancer

Characteristics	CD68^+^ TAMs		*P* value	CD163^+^ TAMs		*P* value	CD66b^+^ TANs		*P* value
	CD68‐low	CD68‐high		CD163‐low	CD163‐high		CD66b‐low	CD66b‐high	
Case No.	331	331		331	331		331	331	
Sex, *n* (%)									
Male	231 (69.8)	245 (74.0)	0.226^a^	233 (70.4)	243 (73.4)	0.387^a^	239 (72.2)	237 (71.6)	0.863^a^
Female	100 (30.2)	86 (26.0)		98 (29.6)	88 (26.6)		92 (27.8)	94 (28.4)	
Age, *n* (%)									
≤60	200 (60.4)	170 (51.4)	**0.019** a	198 (59.8)	172 (52.0)	**0.042** a	192 (58.0)	178 (53.8)	0.273^a^
>60	131 (39.6)	161 (48.6)		133 (40.2)	159 (48.0)		139 (42.0)	153 (46.2)	
Tumor size (cm), *n* (%)									
≤5.0	254 (76.7)	225 (68.0)	**0.012** a	264 (79.8)	215 (65.0)	**<0.001** a	210 (63.5)	269 (81.3)	**<0.001** a
>5.0	77 (23.3)	106 (32.0)		67 (20.2)	116 (35.0)		121 (36.5)	62 (18.7)	
Differential grade, *n* (%)									
Well	10 (3.0)	4 (1.2)	**<0.001** b	13 (3.9)	1 (0.3)	0.435^b^	6 (1.8)	8 (2.4)	**<0.001** b
Moderate	90 (27.2)	106 (32.0)		86 (26.0)	110 (33.2)		77 (23.3)	119 (36.0)	
Poor	231 (69.8)	221 (66.8)		232 (70.1)	220 (66.5)		248 (74.9)	204 (61.6)	
TNM stage, *n* (%)									
I	127 (38.4)	75 (22.7)	**<0.001** b	130 (39.3)	72 (21.8)	**0.001** b	81 (24.5)	121 (36.6)	**<0.001** b
II	86 (26.0)	94 (28.4)		86 (26.0)	94 (28.4)		87 (26.3)	93 (28.1)	
III	113 (34.1)	156 (47.1)		111 (33.5)	158 (47.7)		154 (46.5)	115 (34.7)	
IV	5 (1.5)	6 (1.8)		4 (1.2)	7 (2.1)		9 (2.7)	2 (0.6)	
Adjuvant chemotherapy, *n* (%)									
Yes	209 (63.1)	249 (75.2)	**0.001** a	215 (65.0)	243 (73.4)	**0.018** a	238 (71.9)	220 (66.5)	0.130^a^
No	122 (36.9)	82 (24.8)		116 (35.0)	88 (26.6)		93 (28.1)	111 (33.5)	
Serum CEA,*n* (%)									
<5 ng/mL	265 (80.1)	248 (74.9)	0.129^a^	272 (82.2)	241 (72.8)	**0.003** a	249 (75.2)	264 (79.8)	0.191^a^
≥5 ng/mL	49 (14.8)	63 (19.1)		42 (12.7)	70 (21.2)		62 (18.7)	50 (15.1)	
Missing	17 (5.1)	20 (6.0)		17 (5.1)	20 (6.0)		20 (6.1)	17 (5.1)	
Serum CA199,*n* (%)									
<37 U/mL	264 (79.8)	245 (74.0)	0.228^a^	265 (80.0)	244 (73.7)	0.396^a^	256 (77.3)	253 (76.4)	0.730^a^
≥37 U/mL	40 (12.1)	49 (14.8)		42 (12.7)	47 (14.2)		43 (13.0)	46 (13.9)	
Missing	27 (8.1)	37 (11.2)		24 (7.3)	40 (12.1)		32 (9.7)	32 (9.7)	

TAM, tumor‐associated macrophages; TAN, tumor‐associated neutrophils; CEA, carcinoembryonic antigen; CA19‐9, carbohydrate antigen 19‐9; TNM, tumor node metastasis, the bold emphasizes when *P* < 0.05.

a Chi‐square test or Fisher's exact test.

b Mann–Whitney U test (nonparametric).

### Prognostic values of individual tumor‐infiltrating immune cells

**Figure 2:**
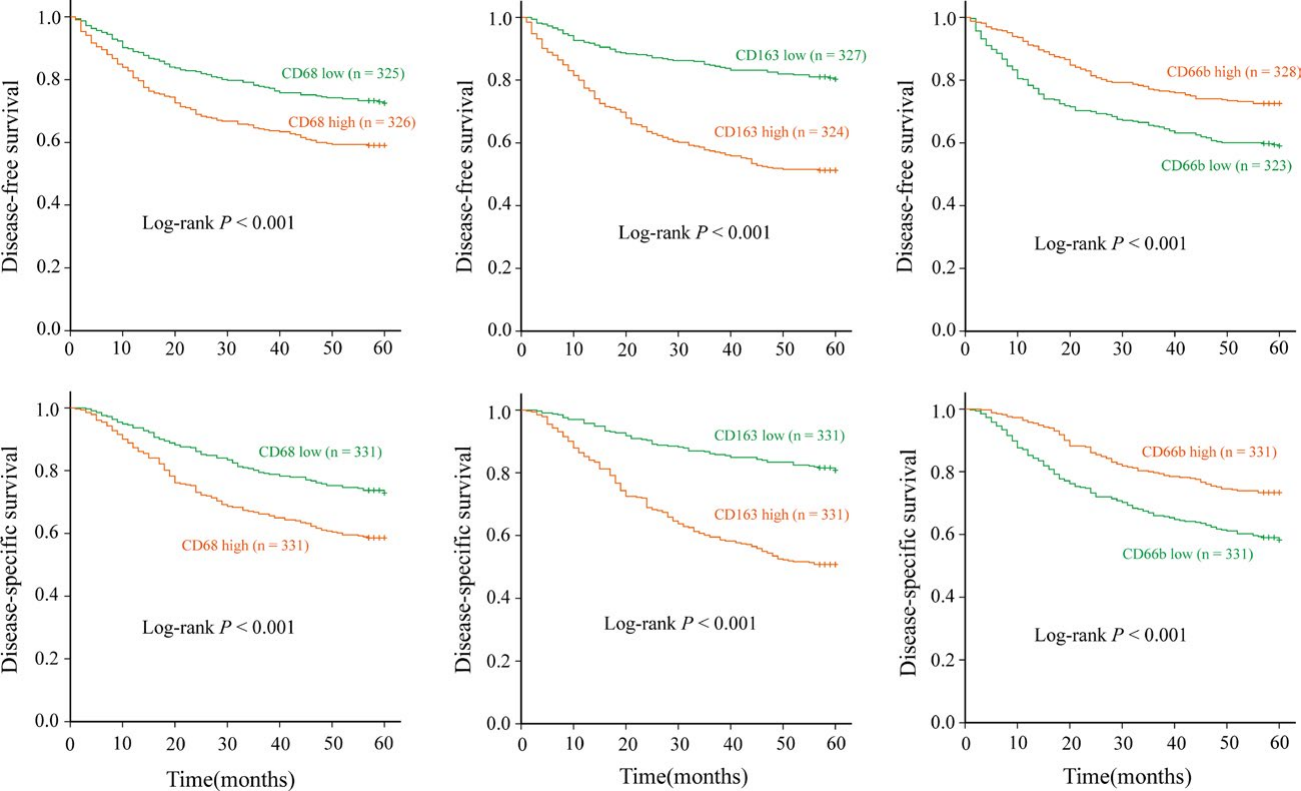
Associations between individual immune cell populations marked with CD68, CD163, or CD66b and patient survival outcomes. Disease‐free survival (A) or disease‐specific survival (B) with high (red line) or low (green line) infiltration of CD68, CD163, or CD66b in GC specimens.

Survival analysis with Kaplan–Meier and log‐rank tests showed that the infiltration of each of immune cells marked with CD68, CD163, and CD66b was associated with patient survivals. As shown in Figure 2, high infiltration of CD68^+^ or CD163^+^ macrophages was associated with short DFS and DSS (all *P *<* *0.001), whereas high infiltrating CD66b^+^ neutrophils were significantly associated with long DFS and DSS (all *P *<* *0.001). Considering the effects of each immune cell and the clinicopathological features, including TNM stage and differentiation grades, on the prognosis of GC, CD68^+^ (hazard ratio (HR)  = 1.405; 95% confidence interval (CI)  = 1.059–1.866; *P* = 0.019), CD163^+^ (HR = 2.483; 95% CI = 1.824–3.380; *P *<* *0.001), or CD66b^+^ cells (HR = 0.730; 95% CI = 0.543–0.982; *P* = 0.038) were independently associated with DFS. Similar associations were obtained for these markers with DSS (for CD68^+^ cells: HR = 1.424; 95% CI = 1.070–1.896; *P* = 0.015; for CD163^+^ cells: HR = 2.546; 95% CI = 1.863–3.479; *P* = 0.001; for CD66b^+^ cells: HR = 0.729; 95% CI = 0.540–0.984; *P* = 0.039). To exclude the confounding effect of TNM stages on prognosis, we investigated the associations among patient subgroups with different TNM stages. Kaplan–Meier plots showed that high infiltration of CD163^+^ TAMs predicted shorter DFS and DSS only at stage II and III GC but not at stage I GC. However, high CD68^+^ TAMs or low CD66b^+^ TANs predicted shorter DFS and DSS only at stage III GC. The results are shown in Figures [Link], [Link], [Link].

### Construction of a two‐marker model containing CD163^+^ TAMs and CD66b^+^ TANs

To develop a combined model for the prognosis of patients with GC, we initially evaluated the association between the infiltration of CD68^+^, CD163^+^, and CD66b^+^ cells and patient prognosis using multivariate Cox analysis. As expected, the infiltration of CD163^+^ TAMs and CD66b^+^ TANs was remained as independent factors consistently for DFS and DSS, but the infiltration of CD68^+^ cells was excluded (Table S1), indicating that CD163^+^ TAMs were a better predictor of prognosis than CD68^+^ TAMs. Therefore, we combined the infiltration of CD163^+^ TAMs and CD66b^+^ TANs as a two‐marker predictor, which classified 662 patients into four subgroups: CD66b^high^CD163^high^, CD66b^high^CD163^low^, CD66b^low^CD163^high^, and CD66b^low^CD163^low^.

### Prognostic value of the two‐marker predictor in GC

**Figure 3:**
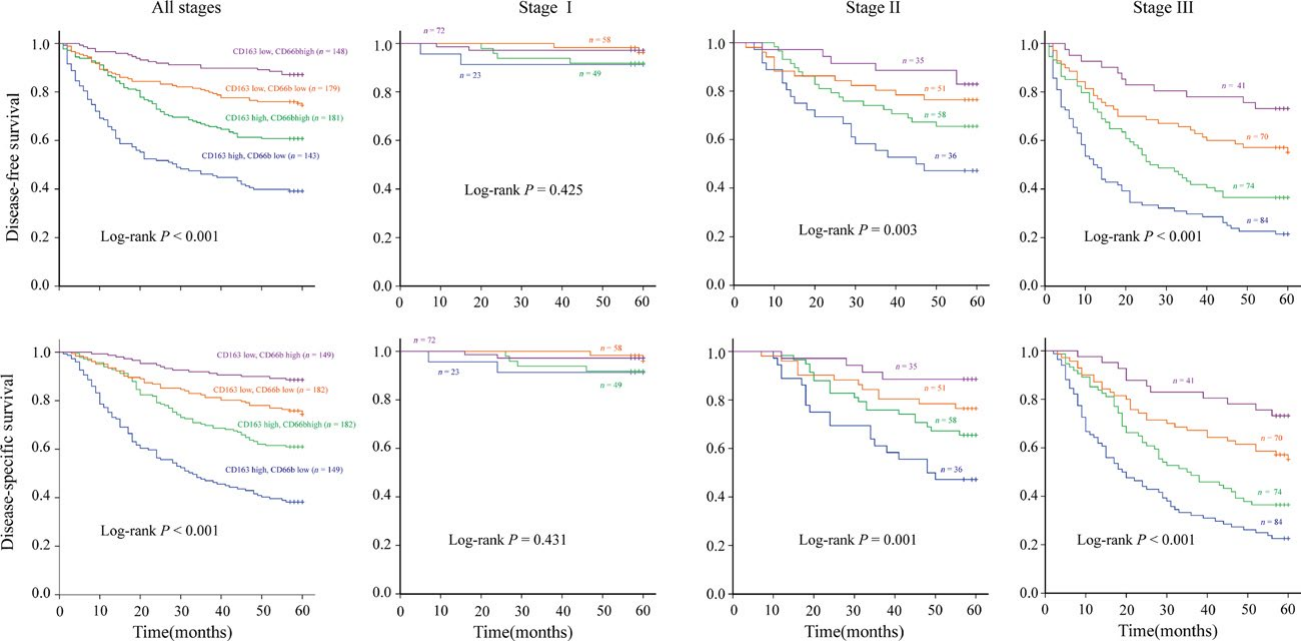
Prognostic values of the two‐marker predictor in all patients or patients with different TNM stages. (A) Kaplan–Meier curves for DFS among subgroups identified by the combination of CD163 and CD66b marked cells; (B) Kaplan–Meier curves for DSS among subgroups identified by the combination of CD163 and CD66b marked cells. The lines highlighted for the subgroups with CD163^low^CD66b^high^ (purple line), CD163^low^CD66b^low^ (red line), CD163^high^CD66b^high^ (green line), and CD163^high^CD66b^low^ (blue line).

As shown in Figure 3, the two‐marker predictor can discriminate the survival outcomes (DFS and DSS) with higher resolution than any individual markers among the 662 patients with GC. Patients with CD66b^low^CD163^high^ had the shortest DFS and DSS among all four subgroups, whereas patients with CD66b^high^CD163^low^ had the longest DFS and DSS. Several tumor aggressiveness features, including tumor size (*P *<* *0.001), TNM stage (*P *<* *0.001), differentiation grades (*P* = 0.004), and serum CEA (*P *< 0.001), were also significantly associated with patient subgroups classified by the two‐marker classifier, as shown in Table 2. Univariate Cox analysis showed that patient subgroups with CD66b^low^CD163^low^ (hazard ratio (HR)  = 2.161; 95% confidence interval (CI)  = 1.266–3.688; *P *<* *0.001), CD66b^high^CD163^high^ (HR = 3.575; 95% CI = 2.155–5.933; *P *<* *0.001), and CD66b^low^CD163^high^ (HR = 7.514; 95% CI = 4.583–12.312; *P *<* *0.001) were gradually associated with shorter DFS when compared with the subgroup with CD66b^high^CD163^low^ (Table 3). Multivariate Cox analysis showed that the two‐marker classifier could independently predict the prognosis of GC with gradually increased HR values when the CD66b^high^CD163^low^ subgroup was used as a reference: CD66b^high^CD163^high^ (HR = 1.887; 95%CI=1.042‐3.415; *P* < 0.001), CD66b^low^CD163^low^ (HR = 3.151; 95% CI = 1.782‐5.571; *P* < 0.001), and CD66b^low^CD163^high^ (HR = 4.945; 95% CI = 2.796‐8.745; *P* < 0.001), as shown in Table 3, together with the covariates, including TNM stages and differentiation grades.

**Table 2:** Association between the infiltration of CD163^+^ TAMs combined with CD66b^+^ TANs and clinicopathologic features in patients with GC

Characteristics	*n*	CD163(A) and CD66b (B)				*P* value^a^
		A^low^B^high^	A^low^B^low^	A^high^B^high^	A^high^B^low^	
Number, *n* (%)	662	149 (22.5)	182 (27.5)	182 (27.5)	149 (22.5)	
Gender, *n* (%)						
Male	476	109 (73.2)	124 (68.1)	128 (70.3)	115 (77.2)	0.300
Female	186	40 (26.8)	58 (31.9)	54 (29.7)	34 (22.8)	
Age (years), *n* (%)						
≤60	370	87 (58.4)	111 (61.0)	91 (50.0)	81 (54.4)	0.172
>60	292	62 (41.6)	71 (39.0)	91 (50.0)	68 (45.6)	
Tumor size (cm), *n* (%)						
≤5	479	133 (89.3)	131 (72.0)	136 (74.7)	79 (53.0)	**<0.001**
>5	183	16 (10.7)	51 (28.0)	46 (25.3)	70 (47.0)	
Differential grade, *n* (%)						
Well	14	7 (4.7)	6 (3.3)	1 (0.5)	0 (0)	**0.004** b
Moderate	196	48 (32.2)	38 (20.9)	71 (39.0)	39 (26.2)	
Poor	452	94 (63.1)	138 (75.8)	110 (60.5)	110 (73.8)	
TNM stage, *n* (%)						
I	202	72 (48.3)	58 (31.9)	49 (26.9)	23 (15.4)	**<0.001** b
II	180	35 (23.5)	51 (28.0)	58 (31.9)	36 (24.2)	
III	269	41 (27.5)	70 (38.5)	74 (40.7)	84 (56.4)	
IV	11	1 (0.7)	3 (1.6)	1 (0.5)	6 (4.0)	
Chemotherapy, *n* (%)						
Yes	458	93 (62.4)	122 (67.0)	127 (69.8)	116 (77.9)	**0.031**
No	204	56 (37.6)	60 (33.0)	55 (30.2)	33 (22.1)	
CEA (ng/mL), *n* (%)						
<5	513	128 (85.9)	144 (79.1)	136 (74.7)	105 (70.5)	**0.010**
≥5	112	15 (10.1)	27 (14.8)	35 (19.2)	35 (23.5)	
Missing	37	6 (4.0)	11 (6.1)	11 (6.1)	9 (6.0)	
CA199 (U/mL), *n* (%)						
<37	509	120 (80.5)	145 (79.7)	133 (73.1)	111 (74.5)	0.744
≥37	89	18 (12.1)	24 (13.2)	28 (15.4)	19 (12.8)	
Missing	64	11 (7.4)	13 (7.1)	21 (11.5)	19 (12.7)	

CEA, carcinoembryonic antigen; CA19‐9, carbohydrate antigen 19‐9; TNM, tumor node metastasis, the bold emphasizes when *P* < 0.05.

a chi‐square test or Fisher's exact test.

b Mann–Whitney U test (nonparametric).

**Table 3:** Cox regression analysis of cell infiltrations based on two‐marker and clinical‐pathological features with survivals in 662 patients with gastric cancer

Variables	Disease‐free survival				Disease‐specific survival			
	Univariate analysis		Multivariate analysis		Univariate analysis		Multivariate analysis	
	HR (95% CI)	*P* value	HR (95% CI)	*P* value	HR (95% CI)	*P* value	HR(95% CI)	*P* value
CD163(A)and CD66b(B)								
A^low^B^high^	1		1		1		1	
A^high^B^low^	7.514 (4.583–12.312)	**<0.001**	4.522 (2.629–7.777)	**<0.001**	8.298 (4.941–13.933)	**<0.001**	4.945 (2.796–8.745)	**<0.001**
A^high^B^high^	3.575 (2.155–5.933)	**<0.001**	2.803 (1.629–4.823)	**<0.001**	4.003 (2.357–6.796)	**<0.001**	3.151 (1.782–5.571)	**<0.001**
A^low^B^low^	2.161 (1.266–3.688)	**<0.001**	1.700 (0.964–2.998)	0.067	2.404 (1.378–4.193)	**0.002**	1.887 (1.042–3.415)	**<0.001**
Gender								
Female vs. male	1.034 (0.776–1.379)	0.818			1.056 (0.792–1.409)	0.709		
Age(years)								
>60 vs. ≤60	1.284 (0.991–1.664)	0.059			1.254 (0.966–1.627)	0.089		
Tumor size(cm)								
>5 vs. ≤5	3.661 (2.822–4.749)	**<0.001**	1.844 (1.359–2.504)	**<0.001**	3.671 (2.825–4.770)	**<0.001**	1.851 (1.358–2.521)	**<0.001**
Differential grade								
Well vs. Moderate vs. Poor	0.689 (0.523–0.907)	**0.008**			0.687 (0.521–0.906)	**0.008**		
TNM stage								
IV vs. III vs. II vs. I	2.931 (2.441–3.520)	**<0.001**	2.210 (1.758–2.779)	**<0.001**	2.947 (2.450–3.546)	**<0.001**	2.194 (1.739–2.769)	**<0.001**
Adjuvant chemotherapy								
No vs. Yes	0.331 (0.230–0.477)	**<0.001**			0.327 (0.226–0.473)	**<0.001**		
CEA(ng/mL)								
≥5 vs*. *<* *5	1.753 (1.286–2.389)	**<0.001**			1.785 (1.308–2.434)	**<0.001**		
CA199(U/mL**)**								
≥37 vs*. *<* *37	1.885 (1.353–2.624)	**<0.001**			1.930 (1.385–2.689)	**<0.001**		

HR, hazard ratio; CI, confidence interval; TNM, tumor node metastasis, the bold emphasizes when *P* < 0.05.

### Two‐marker predictor indicated survival in multiple patient subgroups

**Figure 4:**
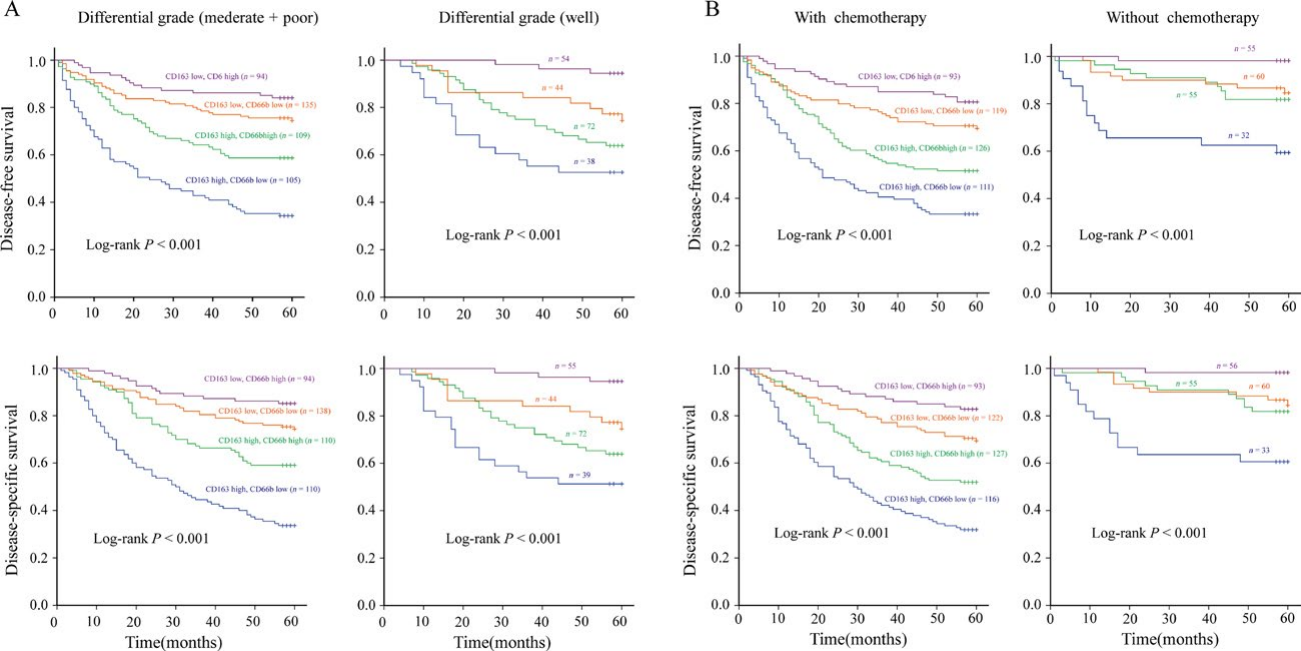
Prognostic values of the two‐marker predictor in patients with different differentiation status or different chemotherapy regimens. (A) Kaplan–Meier curves by two‐marker predictor for DFS and DSS among patients with different differentiation status; (B) Kaplan–Meier curves by two‐marker predictor for DFS and DSS among patients with or without chemotherapy. The lines highlighted for the subgroups with CD163^low^CD66b^high^ (purple line), CD163^low^CD66b^low^ (red line), CD163^high^CD66b^high^ (green line), and CD163^high^CD66b^low^ (blue line).

The prognostic values of the two‐marker model were further evaluated among different patient subgroups, classified by different TNM stages, differentiation grades, and treatment regimens. The survival analyses in each of the TNM stages revealed that the two‐marker model can significantly discriminate the differences in DFS and DSS for patient subgroup with either stage II or stage III GC (Fig. 3). Confined to patients with different differentiation status, the survival analysis revealed that the two‐marker model could significantly discriminate the differences of DFS and DSS for the subgroup with either poor and moderate differentiation or well differentiation (Fig. 4A). Considering the regimens with or without postoperative chemotherapy, we found the two‐marker model could significantly discriminate the differences of DFS and DSS (Fig. 4B); however, the model discriminated the prognosis among the patients with chemotherapy more effectively than that among those without chemotherapy (*P *<* *0.05).

## Discussion

Mixed immune cell populations and their polarizations, such as M1 or M2 TAMs and N1 or N2 TANs, play critical roles in the progression of GC 22, 23, 24, 25. However, their interactions and corresponding clinical significance remain elusive. In the present study, we detected the infiltration of CD68^+^ TAMs, CD163^+^ TAMs, and CD66b^+^ TANs in 662 cases of GC tissues by immunohistochemistry and analyzed the correlations between these cells and their individual and combined prognostic values.

Our results revealed that the infiltration of CD163^+^ TAMs was strongly positively correlated with that of CD68^+^ TAMs, and high infiltrations of CD68^+^ or CD163^+^ TAMs were associated with aggressive characteristics of tumors and an independent poor factor for patients with GC. However, the infiltration of CD163^+^ TAMs was a better predictor than that of CD68^+^ TAMs in the present study, for CD163^+^ TAMs represented a more specific function (M2 macrophage), whereas CD68^+^ TAMs were a mixed population, including protumor or antitumor cells, which reduced the prognosis value of CD68 as a macrophage marker. Many studies have also indicated that high infiltration of CD163^+^ TAMs was correlated with disease progression and poor survival, and thus, it could serve as a poor prognostic marker in GC 2, 13, 26, consistent with the present results. The reversion from M2‐like TAMs to M1‐like cells has been reported when TAMs received IFN‐γ treatment 27, 28, which suggest new possible therapeutic strategies targeting the re‐education of TAMs.

Although neutrophils comprising CD66b have been identified as poor prognostic factors in many types of cancers, including renal cell carcinoma and hepatocellular carcinoma 19, 29, however, these cells have previously been associated with good prognosis in GC 14. The present study also revealed that high infiltration of CD66b^+^ TANs was correlated with favorable tumor characteristics and could serve as an independent good prognostic factor in human GC. Previous studies have shown that the intratumoral‐infiltrated neutrophils undergo polarization toward a protumor or an antitumor phenotype depending on certain environment signals 11. Thus, TANs may have quite varied effects on tumor cells. The blockade of transforming growth factor β (TGF‐β) induced a transformation from the tumor‐promoting type to an antitumor phenotype, suggesting a classification scheme for TANs similar to the M1/M2 phenotype of TAMs 9, 30. High infiltration of CD66b‐marked TANs in GC tissues correlated with a good prognosis in the present study and also indicated that N1 phenotype neutrophils may be the predominant cell type in the GC tissues, although there are no specific markers that can be used to distinguish N1/N2 subgroups. Therefore, the precise infiltration profiles of N1/N2 in GC, their functional roles, and underlying molecular mechanisms need to be further investigated.

When combined them for survival analysis, we found that low infiltration of CD163^+^ TAMs combined with high infiltration of CD66b^+^ TANs showed the longest DSS and DFS, sequentially followed by CD66b^low^CD163^low^, CD66b^high^CD163^high^, and CD66b^low^CD163^high^, indicating that the antitumor ability of CD66b^+^ neutrophils may be overwhelming tumor‐promoting CD163^+^ macrophages. The multivariate analysis revealed that high infiltration of CD163^+^ TAMs combined with low infiltration of CD66b^+^ TANs was an independent poor prognostic factor for patients with GC. The incorporation of the infiltration of CD163^+^ TAMs and CD66b^+^ TANs into the TNM staging system could more significantly predict the prognosis of GC clinically at certain tumor stage. Moreover, the combined predictor could discriminate the prognosis of GC more effectively among the patients with chemotherapy than among those without chemotherapy. These results need larger, more prospective, and multicentered data to validate.

The biological research about the interactions between TANs and TAMs is limited. A few studies have revealed that neutrophils may recruit macrophages and promote the M2‐like activation of macrophages in the tumor microenvironment 11. Besides, some researchers inferred that neutrophils may also directly educate macrophages through myeloperoxidase (MPO) and macrophage mannose receptor (MMR) signaling 31, 32, 33, during which MPO is secreted by neutrophils and MMR is expressed on M2‐like macrophages. In addition, macrophages may be able to resolve the inflammation response induced by neutrophils by removing the dribs of neutrophils 12, 34. Thus, the communication mechanisms between TAMs and TANs are complicated, and further study should focus on the detailed mechanisms about how to control the recruitment and polarization of these two cells, which could identify important targets for anticancer therapies.

In conclusion, individual immune population marked with CD68, CD163, or CD66b was valuable for the prediction of prognosis in GC. However, the infiltration of CD163^+^ cells and CD66b^+^ cells was better prognostic markers than that of CD68^+^ cells. And the infiltration of CD163^+^ TAMs combined with CD66b^+^ TANs could more precisely predict the survival outcomes and could be used as a promising marker for the prognosis of GC.

## Conflict of Interest

The authors declare no potential conflict of interests.

## Supporting information


**Table S1**. Multivariate Cox analysis of three markers for disease‐free survival and disease‐specific survival in 662 gastric cancer patients.

**Figure S1.** Disease‐free survival (A) or disease‐specific survival (B) with high (red line) or low (green line) expression of CD68 of GC patients at different stages.

**Figure S2.** Disease‐free survival (A) or disease‐specific survival (B) with high (red line) or low (green line) expression of CD163 of GC patients at different stages.

**Figure S3.** Disease‐free survival (A) or disease‐specific survival (B) with high (red line) or low (green line) expression of CD66b of GC patients at different stages.
