# Prognostic significance of the infiltration of CD163^+^ macrophages combined with CD66b^+^ neutrophils in gastric cancer

**DOI:** 10.1002/cam4.1673

**Published:** 2018-07-18

Xiaopei Huang, Yamin Pan, Jun Ma, Zhengchun Kang, Xiaowen Xu, Yan Zhu, Jikuai Chen, Wei Zhang, Wenjun Chang & Jiangbo Zhu

Cancer Medicine 2018; 7: 1731‐1741

In the published version of this article, the authors illustrated that their work was supported by grants from the National Natural Science Foundation of China (NSFC) (8157120886 to JZ, 81372671 and 81572451 to WC). However, the number of NSFC to JZ should be corrected to 81573111. Please see below correct and complete Funding details:

## FUNDING INFORMATION

This work was supported by grants from the National Natural Science Foundation of China (81573111 to JZ, 81372671 and 81572451 to WC) and from the Military Key Natural Science Foundation of China (BWS14J023 to JZ).

The authors apologizes for this error.

Huang X, Pan Y, Ma J, et al. Prognostic significance of the infiltration of CD163^+^ macrophages combined with CD66b^+^ neutrophils in gastric cancer. *Cancer Med*. 2018;7:1731‐1741.

