# Supplementary material for: Prognostic significance of the infiltration of CD163+ macrophages combined with CD66b+ neutrophils in gastric cancer
Source: Cancer Med. 2018 Mar 24;7(5):1731–41. doi: 10.1002/cam4.1420 (PMC5943426; doi:10.1002/cam4.1420)
Supplement: Supplementary file 1 — Table S1. Multivariate Cox analysis of three markers for disease‐free survival and disease‐specific survival in 662 gastric cancer patients. [file CAM4-7-1731-s001.docx]

**Supplementary Table 1. Multivariate Cox analysis of three markers for disease-free survival and disease-specific survival in 662 gastric cancer patients**

| **Variable** | **DFS** | |  | **DSS** | |
| --- | --- | --- | --- | --- | --- |
|  | **HR (95% CI)** | ***P* value** |  | **HR (95% CI)** | ***P* value** |
| **CD68 (high *vs.* low)** | 0.973(0.707-1.339) | 0.868 |  | 0.966 (0.700-1.333) | 0.299 |
| **CD163 (high *vs.* low)** | 3.547 (2.512-5.009) | **< 0.001** |  | 3.672 (2.589-5.209) | **< 0.001** |
| **CD66b (high *vs.* low)** | 0.480 (0.367-0.629) | **< 0.001** |  | 0.473 (0.361-0.621) | **< 0.001** |
