# Supplementary figures and images for: Prognostic significance of the infiltration of CD163+ macrophages combined with CD66b+ neutrophils in gastric cancer
Source: Cancer Med. 2018 Mar 24;7(5):1731–41. doi: 10.1002/cam4.1420 (PMC5943426; doi:10.1002/cam4.1420)

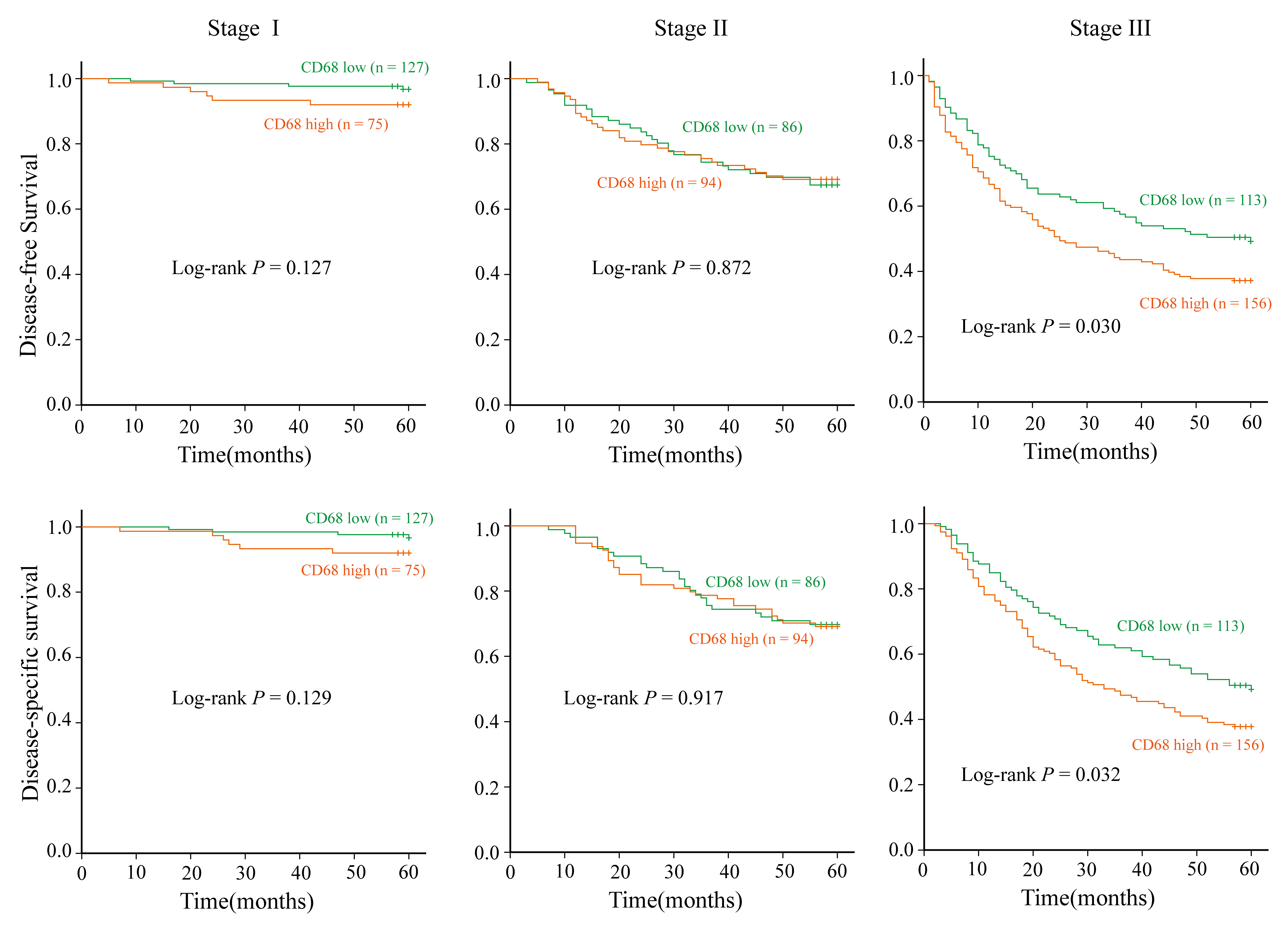

Supplement: Supplementary file 2 — Figure S1. Disease‐free survival (A) or disease‐specific survival (B) with high (red line) or low (green line) expression of CD68 of GC patients at different stages. [file CAM4-7-1731-s002.tif]

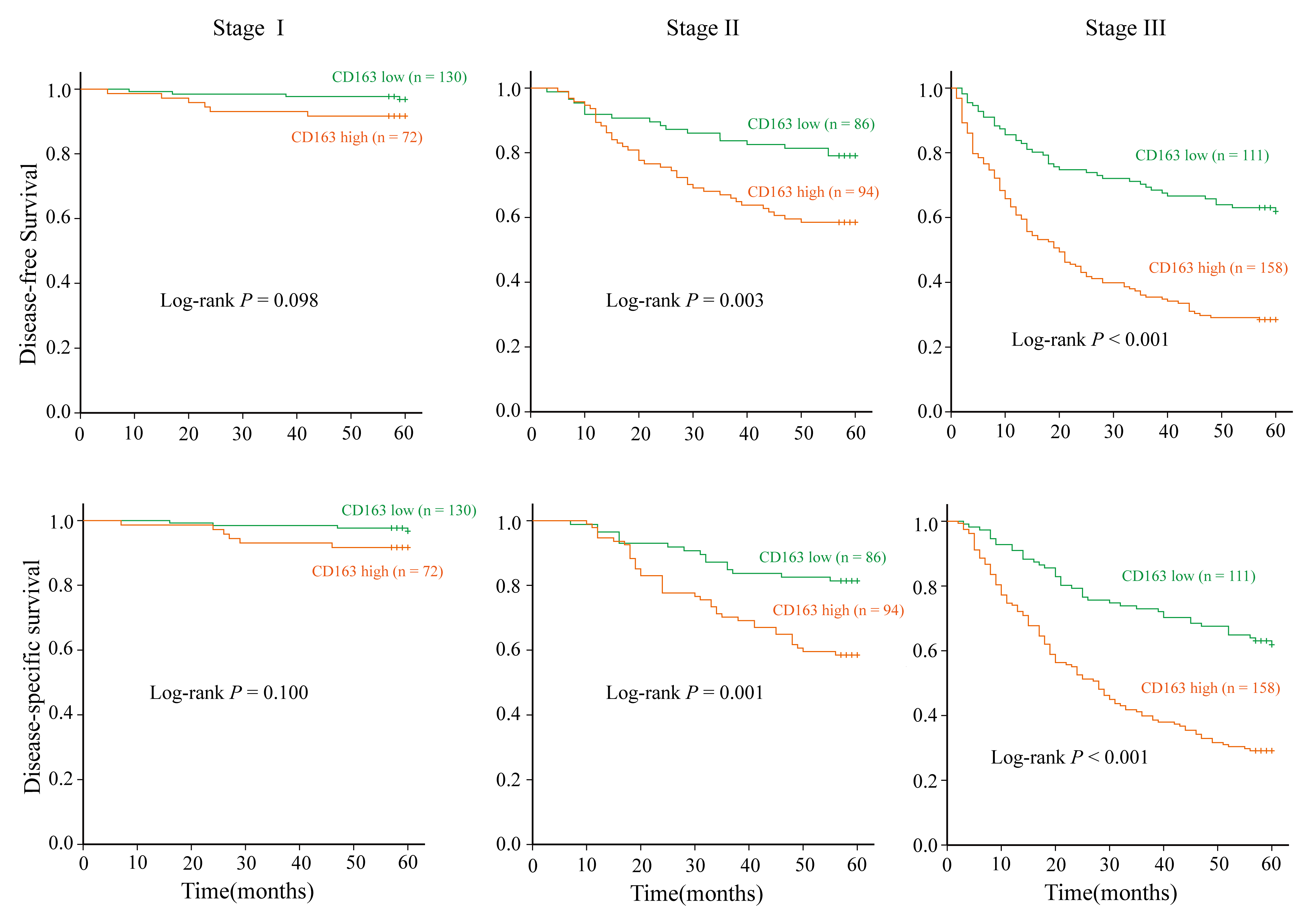

Supplement: Supplementary file 3 — Figure S2. Disease‐free survival (A) or disease‐specific survival (B) with high (red line) or low (green line) expression of CD163 of GC patients at different stages. [file CAM4-7-1731-s003.tif]

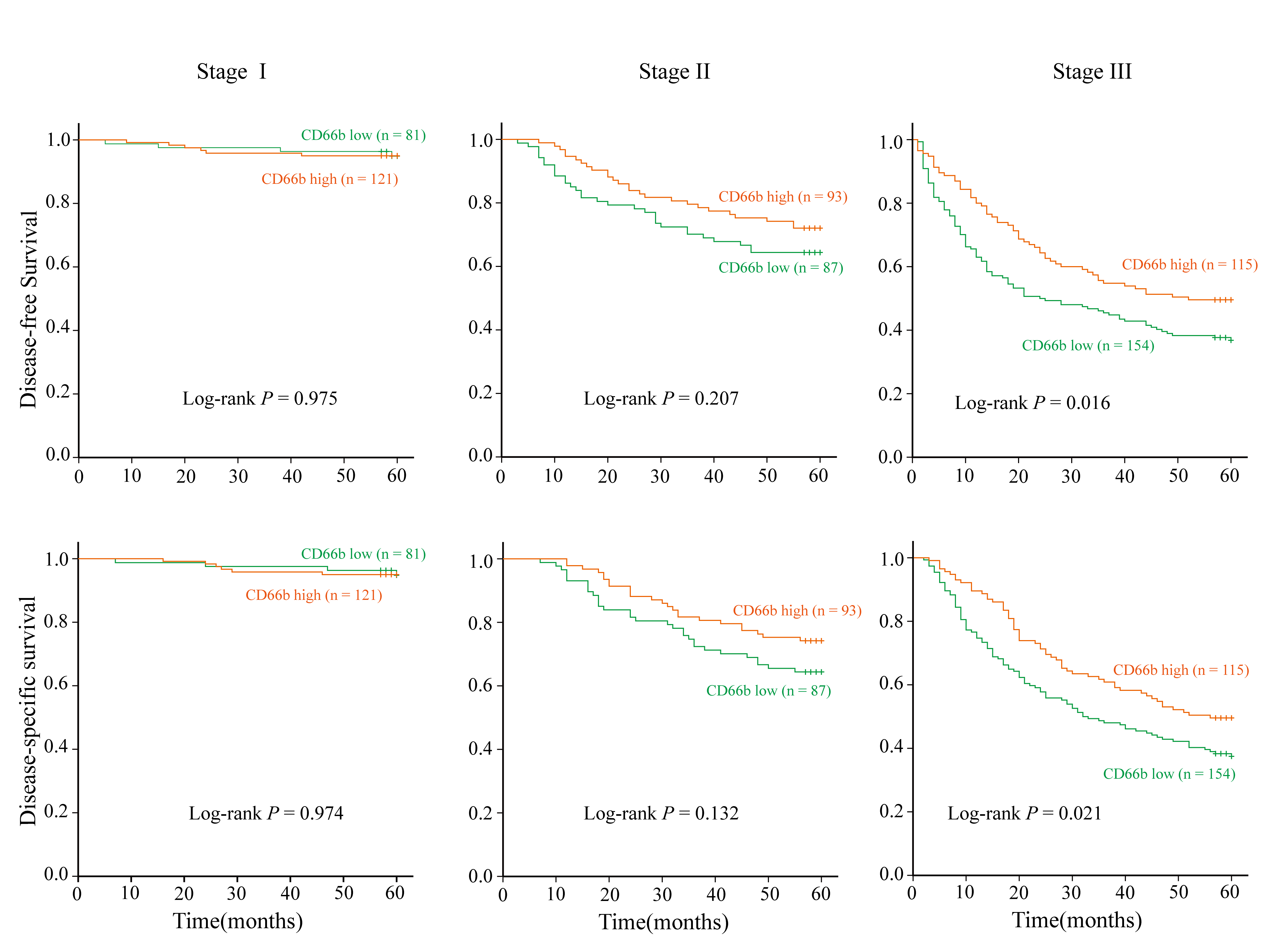

Supplement: Supplementary file 4 — Figure S3. Disease‐free survival (A) or disease‐specific survival (B) with high (red line) or low (green line) expression of CD66b of GC patients at different stages. [file CAM4-7-1731-s004.tif]
